# Association between antihypertensive drugs and the elderly’s oral health- related quality of life: Results of Amirkola cohort study

**DOI:** 10.22088/cjim.13.3.582

**Published:** 2022

**Authors:** Seyyedeh Fatemeh Langari, Seyed Reza Hosseini, Ali Bijani, Niloofar Jenabian, Mina Motalebnejad, Elham Mahmoodi, Zahra Sadat Madani, Fatemeh Sayadi, MohammadMehdi Naghibi Sistani, Reza Ghadimi, Fateme Baladi, Mohammad Hajimirzamohammad, Mahsa Mehryari, Atena Shirzad

**Affiliations:** 1 Student Research Committee, School of Dentistry, Babol University of Medical Sciences, Babol,Iran; 2 Social Determinants of Health Research Centre, Health Research Institute. Babol University of Medical Sciences, Babol, Iran.; 3 Department of Periodontics, Oral Health Research Center, Health Research Institute, Babol University of Medical Sciences, Babol, Iran.; 4 Department of Oral and Maxillofacial Medicine, Oral Health Research Center, Health Research Institute, Babol University of Medical Sciences, Babol, Iran.; 5 Department of Endodontics, Dental Materials Research Center, Health Research Institute, Babol University of Medical Sciences, Babol, Iran; 6 Assistant Professor, Department of Community Oral Health Oral Health Research Center, Health Research Institute, Babol University of Medical Sciences, Babol, Iran.

**Keywords:** Quality of life, Xerostomia, Elderly, Anti-hypertensive drugs

## Abstract

**Background:**

Hypertension is one of the most common chronic diseases in the world. The prevalence of hypertension in the elderly is increasing. Patients with high blood pressure have to take medication throughout their lives. In this study, the relationship between antihypertensive drugs and oral health-related quality of life in the elderly was evaluated.

**Methods:**

This modified cross-sectional study, which is the part of the second phase of the Amirkola Health and Ageing Project (AHAP), was performed on 900 elderly people. Participants included 300 people with hypertension under medical treatment, 300 people with hypertension without medication and 300 people with normal blood pressure. All patients’ blood pressure was recorded, and the standard xerostomia questionnaire and GOHAI questionnaire (Geriatric Oral Health Assessment Index) was completed for all participants. Then, the obtained data were analyzed by SPSS 17, whereby student’s t-test, ANOVA and chi square, Pearson correlation coefficient and logistic regression model were used. A p<0.05 was considered statistically significant.

**Results:**

The mean GOHAI score in the three studied groups: hypertensive under medication treatment, hypertensive without medication treatment and normal blood pressure (51.1±7.4, 51.7±7.3, 51.1±7.5, respectively) did not differ significantly (P=0.533).The frequency of xerostomia was significantly different in the three groups (P=0.008). Among the antihypertensive drugs, the highest rate of xerostomia was due to the use of calcium channel blockers (31.1%) and diuretics (26.8%).

**Conclusion:**

In our study, although antihypertensive medications were associated with xerostomia, they did not decrease the oral health-related quality of life.

Today, hypertension is one of the most important health problems in the world. The prevalence of hypertension in the world in 2010 was reported to be 31.1% (1.4 billion people) and this rate is increasing (1, 2). Premature death, stroke and coronary heart disease are the most important high blood pressure complications (3, 4). Because age is one of the risk factors for high blood pressure, older people are more likely to have hypertension than other age groups. According to studies, 25% of Iranians suffer from high blood pressure, and this rate is 42% in the elderly, higher than in other age groups (5). Advances in blood pressure control have led to the production of a variety of antihypertensive drugs. However, half of people with hypertension do not take medication (6). Today, one of the most important issues is the study of oral health and quality of life-related to oral health in chronic diseases such as hypertension.

Hypertension and antihypertensive drugs can also affect oral health, including hyposalivation and dry mouth, gingival bleeding, facial nerve paralysis, and nodular gingival overgrowth, the most important of which is dry mouth (7). Xerostomia can significantly impact the oral health-related quality of life (OHRQoL) (8). Quality of life refers to how a person feels satisfied or dissatisfied with essential aspects of his life (9). In general, quality of life varies according to age, gender, as well as cultural differences (10). In assessing the quality of life, OHRQoL is also considered, which is the effect of oral health on performance and mental, psychological and social life of the individual (11, 12). Among the OHRQoL measurement tools, today, the use of GOHAI (Geriatric Oral Health Assessment Index) is the key in examining the relationship between oral diseases and quality of life in the elderly. Numerous studies have shown a reduction in health-related quality of life due to hypertension or the use of antihypertensive drugs (13, 14). However, there are very few studies on the relationship between antihypertensive drugs and OHRQoL, especially in the elderly. According to the importance of assessing the quality of life in the elderly with hypertension, the present study was designed to investigate the association of antihypertensive drugs with OHRQoL in the elderly.

## Methods

**Study population and ethics: **This cross-sectional study is a part of the Amirkola cohort study conducted in 2020. The research was approved by the Ethics Committee of Babol University of Medical Sciences (ethics ode=IR.MUBABOL.HRI.REC.1398.307) and all participants entered the study after obtaining informed consent.

**Sample size and data collection: ** The sample size of the present study consisted of 900 patients. To achieve a 95% confidence level, 80% power and assuming a standard deviation of 8 in each group’s GOHAI score, to find **the difference** **of 2 points,** 251 samples were estimated in each group. To increase the accuracy of the study, 300 people in each group were considered. The inclusion criteria were elderly people aged 60 years and older and the subjects were three groups of elderly people: 1- with hypertension and under medical treatment 2- with hypertension without receiving medication 3- people with normal blood pressure who were among 1616 elderly people participating in Amirkola elderly cohort study North of Iran) were selected. The population of each group was 300 (150 men and 150 women).

Basic and demographic information of all subjects such as age, sex, history of diabetes, edentulous, use of removable partial denture, as well as medications used for hypertension were recorded in the information form. All participants’ blood pressure was measured twice using the Omron M3 Intellisense Upper Arm Blood Pressure Monitor while lying down and standing. Hypertension was considered as mean systolic blood pressure greater than or equal to 140 mmHg or diastolic blood pressure greater than or equal to 90 mmHg (1). Then the status of quality of life-related to oral health was assessed using the GOHAI (Geriatric Oral Health Assessment Index) in three groups. The validity and reliability of the Persian version of this questionnaire have been confirmed by Motallebnejad et al.(15). This questionnaire contains 12 questions that were asked in an interview with the individual about his / her condition in the last three months. The questionnaire includes three areas: 1- physical, 2- social and psychological and 3-pain and discomfort. The answers to 12 questions were recorded in the form of 5 options "never", "rarely", "occasionally", "often" and "always" based on the Likert scale.

The total score for the twelve questions was 12-60, with "never" being given a score of 5 and "always" a score of 1. The lower questionnaire score indicates a higher level of quality of life-related to oral health. All participants were evaluated for the presence or absence of xerostomia using a standard xerostomia questionnaire. This questionnaire includes 9 questions whereby if the answer to five questions was yes, xerostomia was confirmed (16, 17).

**Data analysis: **For data analysis, SPSS 17 was used and quantitative and qualitative variables between groups were compared using student t-test, ANOVA and chi square statistical methods. Pearson correlation coefficient and logistic regression model were used for the role of influencing factors. A p 0.05 was considered statistically significant.

## Results

**Figure 1:**
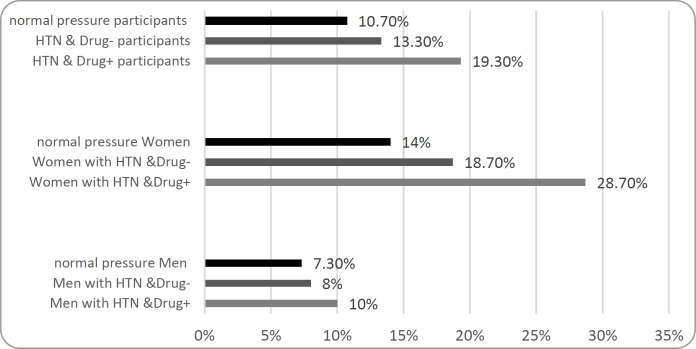
Prevalence of xerostomia in the elderly people

Nine hundred elderly people were included in the study, of which 300 (150 men, 150 women) were hypertensive and were taking antihypertensive drugs (HTN & Drug +), 300 people (150 men, 150 women) had hypertension but did not consume medication (HTN & Drug-) and 300 people (150 men, 150 women) had normal blood pressure (NP). Comparison of age, blood pressure and mean xerostomia and GOHAI score between the three groups are shown in table 1. Out of the 900 subjects, 130 (14.4%) complained of dry mouth, including 16.3% of those with hypertension (with or without antihypertensive drugs) and 10.6% of those with normal hypertension. The frequency of xerostomia was significantly different in the three groups of hypertension under drug treatment, the group hypertension without drug treatment, and individuals with normal blood pressure (P=0.008). Using the Tukey test, this difference was related to two groups with hypertension undergoing drug treatment and individuals with normal blood pressure (P=0.003). This significant difference was true about the three groups of women of the study (P=0.006), but the frequency of xerostomia in the three groups was not significant in the male population (P=0.688) (figure 1).

**Table 1:** Comparison of age, blood pressure and mean xerostomia criteria and mean score of GOHAIα questionnaire between the three groups

**Studied groups** **variable**	**HTN **&Drug+^*^	**HTN **&Drug-^***^	**NP** ^***^	**P- value**
Age (year) Mean±SD	70.6±7.18	70.3±7.55	68.0±6.97	0.0001
Sex				
male n(%) Female n(%)	150(50%) 150(50%)	150(50%) 150(50%)	150(50%) 150(50%)	1
Diabetes melitus n(%)	104(34.7%)	71(23.7)	61(20.3%)	0.00
Removable Partial denture n(%)	170(56.7%)	159(53%)	160(53.3%)	0.6
Edentulous n(%)	140(46.7%)	121(40.3%)	104(34.7)	0.01
Systolic blood pressure (mm Hg) Mean±SD	143.3± 22.8	151.0±16.5	122.6±10.6	0.0001
Diastolic blood pressure (mm Hg) Mean±SD	84.0±11.4	88.8±10.7	76.2±7.6	0.0001
GOHAI^α^ (score) Mean±SD	51.1±7.4	51.7±7.3	51.1±7.5	0.533
xerostomia (score) Mean±SD	2.10±2.6	1.71±2.3	1.48±2.0	0.003

ANOVA Test

^*^^ α^. Geriatric Oral Health Assessment Index

*. (Hypertensive under medication);

**. (Hypertensive without medication);

***. normal pressure

significant

*. Hypertension under medication;

**. Hypertension without medication.

α. Significant

14.4% of the subjects had diabetes. Among the diabetic people, 21.2% and in non-diabetic 12% complained of xerostomia (P=0.001). People with diabetes were twice as likely as non-diabetics to complain of xerostomia (CI95%: 1.3-2.9). 130 out of 900 participants were edentulous (14.4%). The frequency of xerostomia was significantly different between the edentulous and normal groups (17.5% vs. 12.3%, P=0.029). Edentulous people were 1.5 times more likely to have xerostomia than normal people (CI95%:1.0-2.3). Removable partial denture was used by 14.4% of participants. Xerostomia was not significantly different between the two groups with and without removable dentures (P = 0.756, 14.1% vs. 14.8%). The mean of GOHAI score did not differ significantly between the three groups (P=0.533). These results had the same result between men and women separately and the difference between the three groups was not significant.

**Figure 2:**
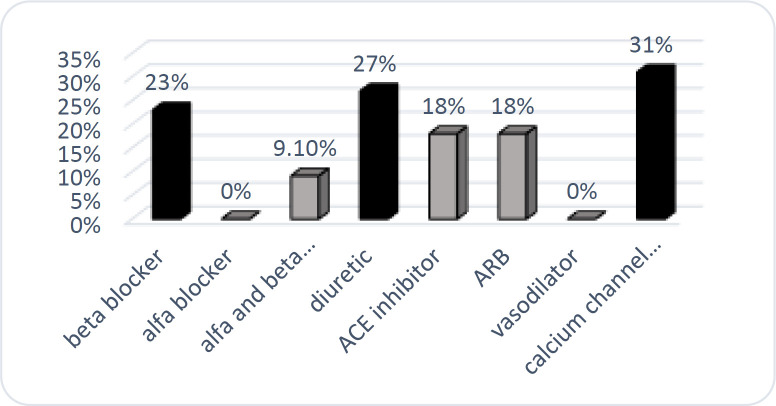
Xerostomia frequency in hypertensive elderly patients receiving antihypertensive drugs

In the current study, the most commonly used antihypertensive drugs were beta-blockers (atenolol-propranolol-metoral), angiotensin receptor inhibitors ARB (losartan-valsartan), calcium channel blockers (amlodipine-nifedipine-diltiazem-verapamil), diuretic (hydrochlorothiazide, furosemide, tiamterene h-spironolactone), ACE inhibitor (captopril, enalapril-lisinopril) and then alpha + beta-blockers (carvedilol) and alpha inhibitors (prazosin-terazosin). The highest rate of xerostomia was associated with calcium channel blockers (31.1%), diuretics (26.8%) and beta-blockers (23.1%), respectively (figure 2). 

**Figure 3:**
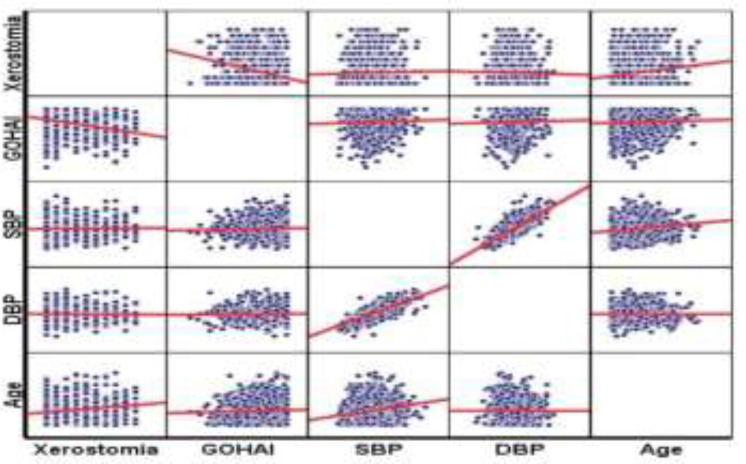
Correlation between oral health related quality of life and other variables: age, systolic blood pressure, diastolic blood pressure and xerostomia

Among the mentioned antihypertensive category drugs, amlodipine, furosemide and propranolol were associated with a higher percentage of xerostomia. Correlation between oral health-related quality of life criteria and other variables: age, systolic blood pressure, diastolic blood pressure, and xerostomia were assessed using the Pearson correlation coefficient. Only xerostomia had a correlation with oral health-related quality of life. The correlation between the two variables was inverse (r=-0.311, P=0.0001), so the people who had xerostomia had a lower quality of life-related to health (figure 3). On the other hand, xerostomia was positively associated with age and number of blood pressure medications used (r=0.166, P=0.0001) and (r=0.123 and P=0.0001). Finally, logistic regression analysis was performed to evaluate the independent factors affecting xerostomia. Older age, female gender, diabetes mellitus and hypertension under drug treatment were independently associated with xerostomia (table 2).

*Pearson correlation test

GOHAI: Geriatric Oral Health Assessment Index, SBP: Systolic Blood pressure, DBP: Diastolic Blood pressure

**Table 2:** Independent variables related to xerostomia based on logistic regression analysis

**P-value**	**Adjusted OR (CI95%)**	**Variable**
0.1	1	Normal pressure
0.651	1.13(0.67-1.88)	HTN &Drug-
0.045^α^	1.65(1.01-2.69)	HTN &Drug+
0.000^ α^	2.94(1.94-4.48)	Sex Female/Male
0.000^ α^	1.06(1.03-1.09)	Age
0.004^ α^	1.85(1.22-2.80)	Diabetes mellitus
0.314	1.27(0.80-2.04)	Edentulous
0.112	0.70(0.44-1.09)	Removable of partial denture

## Discussion

In the present study, which was performed on 900 elderly people in Amirkola city (North of Iran), although the use of antihypertensive drugs was associated with xerostomia, it did not affect the oral health -related quality of life. We did not find a study that directly assessed the relationship between blood pressure medications and oral health-related quality of life. However, the association between hypertension and health-related quality of life has been studied in various studies and showed a reduction people’s quality of life with high blood pressure. In two studies conducted in China in 2017(18) and 2019(19), hypertension was associated with poor life quality. Another study in Pakistan in 2019 found the same result (20). These studies identified higher anxiety and depression, mental disorder and physical inability as the cause of lower quality of life in hypertensive patients compared to normal people.

The relationship between the quality of life and the use of antihypertensive drugs has been evaluated in a few studies. These studies have suggested that physical and mental disorders and poor quality of life in the elderly are the causes of nonadherence to antihypertensive drugs and consequently inadequate blood pressure control (21, 22). People who have a negative attitude towards their health pay less attention to the regular use of their medications (23). Therefore, to optimize blood pressure control, it is recommended to have a psychological support in addition to antihypertensive drugs. In the present study, we found no association between antihypertensive medication use and oral health-related quality of life. In all previous studies, the EQ-5D questionnaire was used to assess the quality of life-related to health, while in the present study, the oral health related quality of life was assessed using a special questionnaire for the elderly, GOHAI, which is a strong tool in this age group. Most people consider oral health disorders as a natural phenomenon in old age and do not look at it as a problem. This attitude can be the cause of not reducing the oral health-related quality of life in this group of people in our society.

Antihypertensive drugs can be associated with xerostomia, and several studies have shown the relationship between xerostomia and reduced oral health-related quality of life, especially in the elderly (24-26). In this study, xerostomia was significantly higher in under medication hypertensive patients than the normal individuals (19.3% vs. 10.7%). In the **Nanzi study in 2012**, as in the current study, the association of antihypertensive drugs with xerostomia was shown. In this study, 50% of people taking blood pressure medications complained of xerostomia versus 25.5% of normal people (27). The xerostomia frequency was higher in the Nanzi study than in the present study. Different xerostomia assessing methods can cause different frequencies.

In our study, a 9-item questionnaire was used, while in the Nanzi study, a Fox et al. questionnaire, which had 5 questions, was used. Given that questionnaires assess the feeling of xerostomia in people, the type of questions and how to ask can affect the frequency of dry mouth. On the other hand, the xerostomia questionnaire is a subjective criterion and may vary according to each society’s race, age and culture. Another reason could be the difference in the type of antihypertensive drugs used by individuals in the two studies.

In the present study, the role of age, sex and diabetes as independent factors in the incidence of xerostomia was evaluated using logistic regression analysis. Similar to other studies (28-30), older age, female gender and diabetes were independently associated with xerostomia. The cause of xerostomia in these groups of people can be due to the greater frequency of systemic diseases and the use of various drugs in old age, autoimmune diseases and hormonal disorders in women and dehydration in diabetes (31).

In addition, calcium channel blockers, diuretics, and beta-blockers were associated with the highest rates of xerostomia. Other studies have reported a correlation between beta-blockers and dry mouth (32). The cause can be due to the activation of the central nervous system and alpha 2-adrenergic receptors of the salivary gland following the use of these drugs (32). 

Diuretics are also drugs that cause xerostomia (33, 34). Similar to other studies, complaints of xerostomia were high in people taking furosemide. Previous studies have reported ten times more oral thrush from furosemide than placebo (35, 36). The reason is the decrease in electrolytes’ output, especially potassium and chloride from the secretions of the submandibular and sublingual glands by furosemide. The association of calcium channel blockers with xerostomia has been shown in previous studies (27, 37). These drugs reduce H2O secretion by blocking Ca2 + channels and thus cause xerostomia (38). The limitation of the present study was that we only used the questionnaire to evaluate dry mouth. Besides, the amount of saliva secretion that can be helpful in a more accurate diagnosis was not evaluated in our study. Also, confounder factors such as systemic diseases, diabetes and edentulous were not matched between groups. However, with the high number of study subjects (900 people) and same sex selection in three groups in addition to the use of logistic regression analysis, the effect of confounder factors was considerably adjusted.

In our study, although antihypertensive medications were associated with xerostomia, they did not decrease the oral health-related quality of life. This result could be due to older people’s culture and attitude towards oral health in our society. 

Therefore, physicians can prescribe antihypertensive drugs without worrying about reducing the oral health-related quality of life in the elderly while making the necessary recommendations, especially the consumption of sufficient fluids to reduce dry mouth. It is suggested that more prospective studies be performed in the elderly with hypertension compared to normal individuals with measurement of salivary secretions and also that all interfering factors be matched.
